# Enhanced Efficiency of Dye-Sensitized Solar Cells Based on Polymer-Assisted Dispersion of Platinum Nanoparticles/Carbon Nanotubes Nanohybrid Films as FTO-Free Counter Electrodes

**DOI:** 10.3390/polym13183103

**Published:** 2021-09-15

**Authors:** Jia-Wun Li, Yu-Sheng Chen, Yan-Feng Chen, Jian-Xun Chen, Chung-Feng Jeffrey Kuo, Liang-Yih Chen, Chih-Wei Chiu

**Affiliations:** 1 Department of Materials Science and Engineering, National Taiwan University of Science and Technology, Taipei 10607, Taiwan; a12352335@gmail.com (J.-W.L.); Chenchen19951221@gmail.com (Y.-S.C.); kk0960216886@gmail.com (Y.-F.C.); ch60210@gmail.com (J.-X.C.); jeffreykuo@mail.ntust.edu.tw (C.-F.J.K.); 2 Department of Chemical Engineering, National Taiwan University of Science and Technology, Taipei 10607, Taiwan; sampras@mail.ntust.edu.tw

**Keywords:** platinum nanoparticles, carbon nanotubes, counter electrodes, dye-sensitized solar cells

## Abstract

In this study, polymer-assisted dispersants are used to stabilize the nanohybrids of platinum nanoparticles (PtNPs)/carbon nanotubes (CNTs) through non-covalent bond forces. These dispersants aim to replace the florine-doped tin oxide (FTO) glass in traditional dye-sensitized solar cells (DSSCs) as counter electrodes. The large specific surface area, high conductivity, and redox potential of PtNPs/CNT nanohybrids are used as the basis to utilize them as the counter electrode material to fabricate a dye-sensitized solar cell. The conductivity results indicate that the resistance of the PtNP/CNT nanohybrid film can be reduced to 7.25 Ω/sq. When carbon nanotubes are mixed with platinum nanoparticles at a weight ratio of 5/1, the photoelectric conversion efficiency of DSSCs can reach 6.28%. When using the FTO-containing substrate as the counter electrode, its conversion efficiency indicates that the micro-/nano-hybrid material formed by PtNPs/CNTs also exhibits an excellent photoelectric conversion efficiency (8.45%) on the traditional FTO substrate. Further, a large-area dye-sensitive cell is fabricated, showing that an 8 cm × 8 cm cell has a conversion efficiency of 7.95%. Therefore, the traditional Pt counter electrode can be replaced with a PtNP/CNT nanohybrid film, which both provides dye-sensitive cells with a high photoelectric conversion efficiency and reduces costs.

## 1. Introduction

The rapid growth of the population on earth and the world economy has brought human demand for energy to an unprecedented level [1]. The large consumption of traditional fossil energy, which leads to resource depletion and environmental pollution [2,3], has also gained increasing awareness [4]. As a result, renewable energy has become the focus of scientific research in recent years [5]. Renewable energy, including hydropower, wind power, tidal energy, geothermal energy, and solar energy, has shown significant development [6,7], among which solar energy has shown great industrial development [8]. Dye-sensitized solar cells (DSSCs) have the advantages of transparency, colorfulness, simplicity in manufacturing, and low production cost, making them one of the most promising devices for solar power generation [9,10]. The main components of a typical DSSC are redox electrolytes (such as iodide/triiodide in an organic solvent), a dye-fixed photoelectrode (PE), and a counter electrode (CE) [11], where an ideal CE should have a high exchange current density and low charge transfer resistance [12]. In the past few decades, various methods have been used to assemble DSSCs [13], for which the counter electrode is one of the key components [14]. To improve the efficiency of dye-sensitized solar cells and reduce the overall cost, the counter electrode has also become the main research subject, due to its simpler manufacturing process [15,16]. Platinum is often used as a counter electrode material because of its excellent catalytic ability [17]. However, it is expensive and has poor stability and, hence, cannot be used in large quantities for commercial mass production [18]. Moreover, the transparent conductive glass such as transparent conductive oxide (TCO) and F-doped tin oxide (FTO) usually comes in the form of a thick hard substrate with expensive coating film, which limits the reduction in the overall cost of DSSCs [19]. Therefore, considerations for cost reduction and future development play a major role in the development of materials that can replace platinum and transparent conductive glass [20].

After the dye-sensitized solar cell was invented in 1991 [21], it has become the focus of many researchers due to its many advantages. However, after several years of research and development by many researchers worldwide, the current laboratory efficiency is still only about 13% [22,23], not to mention the many limitations that need to be overcome in production. In recent years, DSSCs have been developing toward higher stability, higher efficiency, and lower cost [24]. Therefore, manufacturing highly efficient and highly stable DSSCs at a low cost has also become a hot research topic [25]. To reduce the cost and pursue high efficiency simultaneously, it has been reported in recent years that hybrid counter electrodes have been prepared by mixing inorganic substances and platinum to improve the economic efficiency [26,27]. In particular, carbon-containing materials are one of the main focuses of electrochemical research [28]. For example, graphene oxide and TiO_2_ were mixed with Pt to prepare a hybrid counter electrode, with a conversion efficiency of 4.52% [29]. Another study used hexachloroplatinic acid to reduce Pt to the graphene surface through chemical reduction and a highly conductive FTO substrate to prepare a binary composite material with a conversion efficiency of 6.25–8.00% [30,31]. It was also reported that a counter electrode composed of carbon black, and a conductive polymer with conductive glass, can have a conversion efficiency reaching 7.24% [32]. Moreover, due to its high chemical stability, electrical conductivity, thermal conductivity, and excellent mechanical properties [33], multi-walled carbon nanotubes (MWCNTs) have recently been reported on multiple accounts to show good electronic properties in the production of DSSCs [34,35,36]. Samantaray et al. prepared the counter electrode of DSSCs using carbon nanotubes, reporting a conversion efficiency of 7.00–7.81% [37]. Ho et al. used polymer-based organic dispersants to uniformly reduce Pt to MWCNTs, which exhibited an excellent conversion efficiency of 8.00% under the FTO substrate [38]. Liu et al. used similar polymer-based organic dispersants to reduce Pt to the surface of the MWCNTs, without the aid of conductive glass as the counter electrode, reporting a conversion efficiency of 6.96% [39]. In recent studies, there have been several reports on the use of organic dispersants to reduce metal particles into carbon nanomaterials [40,41], but there have not been related papers published on the application of DSSCs.

In this study, four different polymer-assisted dispersants were designed and synthesized, and platinum precursors was used to prepare PtNPs through chemical redox reactions. Polymer-assisted dispersants can disperse the PtNPs uniformly and stably on the surface of CNTs through non-covalent bond forces (such as ion-dipole interaction, hydrophobic effect, lone pair-π stacking), and the hybrid material can improve the photoelectric conversion efficiency of the counter electrode after hybridization. In addition, using carbon nanotubes as the substrate can also reduce the cost of pure platinum materials in the traditional counter electrode. Furthermore, as a one-dimensional carbon nanomaterial, CNTs have excellent electrical conductivity and flexibility, whose high specific surface area can be utilized to absorb platinum nanoparticles, forming a hybrid complex of two different dimensions. This is finally applied to the DSSCs’ counter electrode, which can improve the photoelectric conversion efficiency of the cell.

## 2. Experimental

### 2.1. Materials

Polyisobutylene-g-succinic anhydride (PIB-SA, Mw = 1335) was purchased from Yuang Hong Corp. (Taipei, Taiwan). A series of poly(oxyethylene)-amine and Poly(oxyethylene)-diamine, with the designated trade name of Jeffamine^®^ M1000 (Mw = 1000), Jeffamine^®^ D900 (Mw = 900), Jeffamine^®^ ED2003 (Mw = 2000), and Jeffamine^®^ D2000 (Mw = 2000), were obtained from Huntsman Chemical Co., Los Angeles, CA, USA. Multi-wall carbon nanotube (CNT; diameter: 10–20 nm, length: 10–30 μm, purity: ≥97%, specific surface area: >200 m^2^/g) was obtained from Conjutek Co. Ltd., New Taipei City, Taiwan. Titanium dioxide (TiO_2_) paste, with the designated trade name of P300 (particle size: 20–50 nm) and P400 (particle size: 350–400 nm), was purchased from Ruilong Co. Ltd., Miaoli County, Taiwan. Tetrahydrofuran, hexachloroplatinic acid (H_2_PtCl_6_), sodium borohydride (NaBH_4_), 4-tert-butylpyridine (tBP), lithium iodide (LiI), and guanidine thiocyanate (GuSCN) were purchased from Sigma-Aldrich (St. Louis, MO, USA). Finally, iodine (I_2_) was obtained from Riedel-de Haen, and 1,3-dimethylimidazolium iodide (DMII) was purchased from Uniregion Bio Tech, Taoyuan City, Taiwan.

### 2.2. Synthesis of Polymer-Assisted Dispersants

In this experiment, four organic dispersants were synthesized to investigate the dispersibility of carbon tubes: PIB-M1000 with a molecular weight of 1000 and single branch, PIB-ED900-PIB with a molecular weight of 900 and double branches, PIB-2003-PIB, and PIB-D2000-PIB; the latter two have a molecular weight of 2000 and double branches. The synthesis follows the steps below. The linear oil-soluble organic dispersant polyisobutylene-g-succinic anhydride (PIB-SA) and linear hydrophilic polyetheramine (M1000, ED900, ED2003, D2000) are added to the organic solvent THF at 25 °C with a molar ratio of 1/1 or 2/1 (which depends on whether the monofunctional group or difunctional group is present), which allows for the amination branching reaction to be carried out for 6 h to synthesize poly(isobutylene)-PIB-amide. The resultant solution is dehydrated at a high temperature of 150 °C for 6 h to finally synthesize the linear amphiphilic organic dispersant-poly(isobutylene)-imide (i.e., PIB-imide). The synthesis reactions are shown in Figures S1 and S2 in the Supporting Information. The Fourier transform infrared spectroscopy (FT-IR) is used to identify the synthesis of organic dispersants, as shown in Figure S3. The C=O of PIB-SA (anhydride) shows characteristic peaks at wavenumbers 1780 and 1710 cm^−1^. After 6 h of amidation branching reaction at room temperature, the characteristic peaks of amide appear at wavenumbers 1640 cm^−1^ from C=O and 1540 cm^−1^ from N-H of PIB-amide (M1000). After 6 h of dehydration at 150 °C, the characteristic peaks of C=O of PIB-imide (M1000) appear at wavenumbers 1710 and 1650 cm^−1^. The other three organic dispersants all have corresponding C=O and N-H characteristic peaks. The results of the FT-IR test show that the long-chain polymer-assisted dispersant with hydrophilic and lipophilic segments has been synthesized successfully. To remove the organic solvent THF, the product is concentrated under reduced pressure at 60 °C for 8 h to take out the purified organic dispersant, which is then tested for solubility, as shown in Figure S4 and Table S1. The long carbon chain of polyisobutylene-succinic anhydride (PIB-SA) tends to dissolve in oil phase solvents. For example, low-polarity THF, toluene or non-polar organic solvents, M series, and ED series of polyetheramine all have completely hydrophilic polyoxyethylene (POE) segments. Therefore, PIB-M1000 and PIB-ED2003 are soluble in most polar solvents, whereas PIB-ED900-PIB is less soluble in comparison due to the relatively lower number of POE segments. In contrast, the main segment of the D series of polyetheramines is predominately polypropylene glycol (PPG), which is less hydrophilic, and thus the dispersant PIB-D2000-PIB emulsifies in the water. After amidation and branching, the main structure of the copolymer is composed of PIB-POE-PIB, which is amphiphilic, indicating that it is soluble in both polar solvents and non-polar solvents. Amphiphilicity can be achieved by adjusting the number of POE or PIB segments.

### 2.3. Preparation of Photoanode

The size of the substrate is designed to be 1.5 × 2.0 cm^2^, which is cleaned using the improved RCA cleaning method invented by Werner Kern in 1970 at the Radio Corporation of America. To keep the conductive glass transparent during the cleaning process, the RCA cleaning method is utilized to clean the substrate. The substrate is to be first wiped with anhydrous alcohol wipe to remove dust particles and then soaked in deionized water containing surfactant, pure deionized water, acetone, and isopropanol for 30 min each to remove oil stains and surface organic matter. After the cleaning is completed, the adsorption layer is prepared by coating the P300 titanium dioxide slurry on the conductive surface of the conductive glass. The coating area is controlled with tape, and the coating thickness is set to 2250 μm by a film thickness controller based on the conductive glass thickness of 2.2 mm. The P300 titanium dioxide slurry is evenly coated onto the conductive glass at a set speed of 5 mm/s of the knife coater. After each layer of coating is applied, it is heated at 250 °C with a hot air gun to be smoothened. This procedure is repeated three times, and the coated layers are sintered to 500 °C for 2 h under atmospheric conditions such that the titanium dioxide layer and FTO can form a dense bond and completely transform the phase into anatase phase. Subsequently, the scattering layer is prepared in the same manner as the adsorption layer with P400 slurry containing larger particles. After coating and sintering, the titanium dioxide layers containing the adsorption layer with smaller particles and the scattering layer with larger particles are completed. Following which, N719 of 0.3 mM concentration is added to a mixed solution containing tert-butanol and acetonitrile at a volumetric ratio of 1:1. The annealed titanium dioxide photoanode is put into this N719 solution for 24 h of adsorption to prepare the photoanode.

### 2.4. Preparation of the PtNPs/CNT Hybrid Material

In this experiment, 25 mg of carbon nanotubes were prepared as an aqueous solution with a weight concentration of 0.25 wt.%, into which a polymer-assisted dispersant with a weight ratio of 1:1 to the carbon nanotubes is added. Then, this mixture was pre-dispersed with a probe-type ultrasonic oscillator, with amplitude 10, a pulse-on time of 30 s, and a pulse-off time of 3 s. The mixture was processed for 30 min at a frequency of 37 kHz, and then 210, 105, and 10.5 mg of 10 wt.% hexachloroplatinic acid (H_2_PtCl_6_) solved in alcohol were added, respectively, to prepare the solutions, along with a ratio of carbon nanotubes to platinum as 5/1, 10/1, and 100/1. The dispersant solution was further oscillated for 10 min and placed into an ice bath, while 10.5, 5.25, and 0.525 mL of 0.01 M sodium borohydride were all added gradually to the respective dispersant and stirred under a magnet for 1 h. After the reaction was completed, the liquid was rinsed with deionized water five times to remove the remaining sodium borohydride and then freeze-dried to obtain the PtNP/CNT powder.

### 2.5. Preparation of CNTs, PtNPs/CNT Dispersant, and Counter Electrode, and DSSC Packaging

The CNTs and PtNPs/CNT are mixed with N-methylpyrrolidone in a 0.25 wt.% solution, which is to be pre-dispersed with a probe-type ultrasonic oscillator. At an amplitude of 10, a pulse-on time of 30 s, and a pulse-off time of 3 s, the solution is processed for 10 min at a frequency of 37 kHz. An organic dispersant (PIB-ED2003-PIB) with carbon nanotubes-to-dispersant ratios of 2:1, 1:1, and 1:2 is added into the dispersed solution, respectively, to assist the dispersion and stabilization of inorganic particles. The resultant solution is further oscillated under the same conditions above for 10 min to obtain the CNTs and PtNPs/CNT dispersant liquid. Subsequently, 0.5 mL of the above solution is used to drop-coat the FTO substrate and the FTO-free glass substrate, respectively, and then dried at 110 °C to become film. This process is repeated ten times once the film is dry. Following which, the dispersant is to be removed to reduce the surface resistance. The film is sintered to 350 °C for 1 h under atmospheric conditions to form the ten-layer FTO and FTO-free CNT/PtNPs counter electrodes. The working electrode and the counter electrode are tightly sealed together with 60-micrometer heat sealing glue to form a sandwich-like structure. The electrolyte made of 0.1 M LiI, 0.6 M DMII, 0.03 M I2, 0.1 M GuSCN, and 0.5 M tBP, with a volumetric ratio of acetonitrile to valeronitrile of 85:15, is injected into the pre-drilled holes. Finally, the holes are sealed with heat-resistant tape to complete the packaging of the dye-sensitized solar cell.

### 2.6. Characterization and Instruments

Fourier transform infrared spectroscopy (model (FTS-1000)) is used to monitor the reaction process of SMA-amide. The sample is applied to the KBr thin plate and scanned under infrared light of 500 to 4000 cm^−1^. An FTIR curve is obtained in an average of 32 scans. FE-SEM (JSM-6500F) is used to inspect the surface of the conductive film. The inspected sample is coated with a thin layer of platinum before the photo shooting. The resistance of the conductive film is measured with a four-point probe low resistance tester (Mitsubishi Chemical Corporation, MCP-T600, Tokyo, Japan). A dynamic material testing machine (MTS-370, Sinodynamics Enterprise Co., Ltd, Taipei, Taiwan) is used to perform different tensile strain tests on the conductive film. A multimeter is then used to check the changes in resistance before and after the film strain. A transmission electron microscope (TEM, Zeiss EM 902A, Oberkochen, Germany) is used to observe the adsorption of CNT/PtNPs. A measurement sample solution of mass concentration 1 wt.% is first prepared, which is then deposited onto the carbon-coated copper mesh; vacuum is then applied until dry, and TEM is used to inspect the residue. In addition, the weight loss curve can be obtained by using the TA instrument Q-500 (United States DE, New Castle, DE, USA) for thermogravimetric analysis (TGA), in which 5–8 mg of sample is weighed and heated to 600 °C in nitrogen. The electrocatalytic properties of CNT/PtNPs to the I^−^/I_3_^−^ redox couple are studied using cyclic voltammetry. Same as the previous measurement of CV, platinum (Pt) is used as the counter electrode (CE), saturated silver nitrate (Ag/AgNO_3_) is used as the reference electrode, and CNT/PtNPs is used as the working electrode in the three-electrode system. The electrolyte used is 0.1 M LiCl_4_, 0.01 M LiI, and 1 mM I_2_ in acetonitrile. The CNT/PtNPs of three different ratios are discussed. The current density of the oxidation-reduction potential measured shows the catalytic properties of the electrode to the electrolyte. The photocurrent density (J)-photovoltage (V) curve uses AM 1.5 G to simulate the characteristics of the cell under sunlight, where the AM 1.5 G simulated sunlight is generated by a 150 W A-type solar simulator (model 92250A, Oriel), with an illumination intensity of 100 mW/cm^−2^. The incident light intensity is calibrated by a standard crystalline silicon solar cell (Oriel reference cell, 91, 550 V), and a power meter (Keithley 2400) is used to measure the response of the solar cell.

## 3. Results and Discussion

### 3.1. Influence of Polymer-Assisted Dispersant on the Dispersion of CNTs

**Figure 1:**
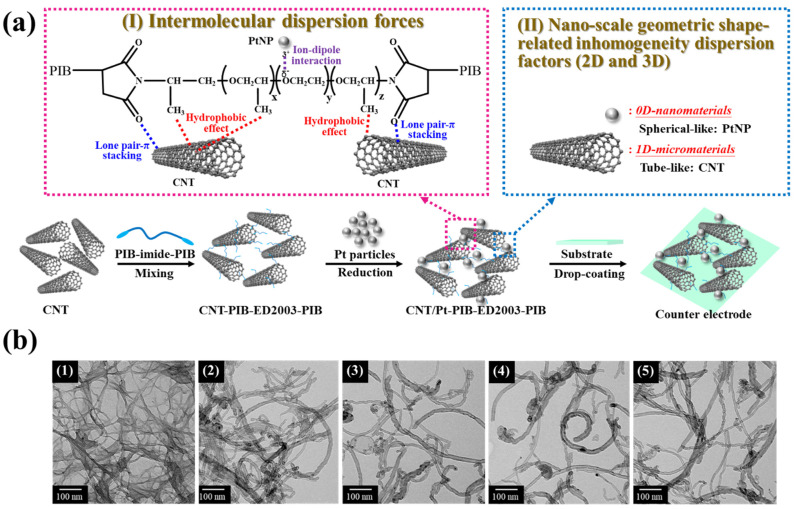
(**a**) Schematic of intermolecular forces between the polymer-assisted dispersant PIB-ED2003-PIB and CNTs and PtNPs. (**b**) TEM diagrams of (1) without dispersant, (2) PIB-M1000, (3) PIB-ED900-PIB, (4) PIB-ED2003-PIB, and (5) PIB-D2000-PIB added at a ratio of 2:1, 1:1, 1:2, and 1:2 to carbon nanotubes.

In this study, four polymer-assisted dispersants were synthesized. The lone pair of electrons on the molecular chain of the dispersant was used to cause the lone-pair interaction between the carbon nanotubes. The hydrophilicity group on the ED2003 chain (OCH_2_CH_2_) and hydrophobicity group on the end of the PIB chain were both adjusted to make the carbon nanotubes more stable in the solvent. Moreover, the ion-dipole interaction formed between O^−^ and Pt^3+^ in the molecular chain of the dispersant was used to ensure that platinum can be more stably reduced around the carbon nanotubes. This phenomenon has already been reported by a previous study [42,43,44], as shown in Figure 1a. To verify the effectiveness of the four dispersants on carbon nanotubes, carbon nanotubes of 0.25 wt.% dispersed in NMP were prepared, with the dispersant-to-carbon nanotubes ratio fixed at 1:1; the mixture was placed under ultrasonic oscillation for 30 min and then left to stand for several days. The macroscopic observation of its dispersion is shown in Figure S5. The single-branch PIB-M1000 and the double-branch PIB-ED900-PIB with similar molecular weights were compared first to assess the effect on the dispersibility of the carbon tubes. It can be observed that on the second day, the carbon tube solution dispersed by PIB-M1000 already shows little precipitation, with aggregated particles sticking to the wall, which becomes much more serious after 20 days. In comparison, the dispersion of carbon nanotubes due to PIB-ED900-PIB is much milder, indicating that the double-branch dispersant has better performance than the single-branch dispersant. Subsequently, the double-branch PIB-ED900-PIB and PIB-ED2003-PIB with different molecular weights were compared for their performance in dispersing carbon nanotubes. It can be observed that, while the PIB-ED2003-PIB with a larger molecular weight shows the stable dispersion of carbon nanotubes on both the second and the 20th day, there are no significant agglomerated particles on the cup wall for the dispersant solution with PIB-ED900-PIB having a smaller molecular weight. This indicates that hydrophilic polyetheramine with a larger molecular weight in the synthetic organic dispersant can effectively help stabilize the carbon nanotubes. Finally, the double-branch organic dispersants PIB-ED2003-PIB and PIB-D2000-PIB with different degrees of hydrophilicity were compared. It can be observed that the less hydrophilic PIB-D2000-PIB causes more precipitation and agglomeration of particles on the cup wall as compared to the more hydrophilic PIB-ED2003-PIB, indicating that changing the polyetheramine segment to adjust the hydrophilic and hydrophobic properties will change the dispersibility of the carbon nanotubes. After this preliminary understanding of the effects of the four dispersants on the dispersion of carbon nanotubes, the penetration of the solution was measured using UV–vis for further confirmation. The dispersion of carbon nanotubes is tested with the specific wavelength of 550 nm through an ultraviolet–vis spectrometer to determine the dispersion effect of the carbon nanotubes since 550 nm wavelength light is most sensitive to human eyes [45]. Thus, Figure S5a,b, respectively, show the transmittance at a 550-nanometer wavelength under different ratios of CNT and dispersant for 2 and 20 days later. When the penetration is lower, it can indicate that the CNTs have no precipitation and are more dispersed in the solution, so that most of the suspended CNTs in the solution absorb the 550-nanometer light and make the penetration lower. When CNT precipitation occurs, the number of suspended CNTs in the solution decreases, resulting in a decrease in light penetration resistance and an increase in 550-nanometer penetration. The results show that single-branch PIB-M1000 and double-branch PIB-ED900-PIB with the same molecular weight of polyetheramine was compared for the UV–vis transmittance, which is 62 for the carbon nanotube solution, without adding the dispersant. This value drops to 56 and 35, respectively, after the dispersant is added at a weight ratio of 1:2. However, after standing for 20 days, this value improves for the two types of dispersant solutions at different weight ratios, up to 67.3 and 55.8, respectively, with a weight ratio of 1:2. This indicates that the carbon nanotubes have precipitated and aggregated. The double-branch PIB-ED2003-PIB is then compared with the double-branch PIB-ED900-PIB, with a different molecular weight, and the double-branch PIB-D2000-PIB with a different degree of hydrophilicity for the dispersion effects on carbon nanotubes. It can be observed that, regardless of the weight ratio, PIB-ED2003-PIB always has a lower UV–vis penetration than PIB-ED900-PIB, which is consistent with the macroscopic observation results, indicating that PIB-ED2003-PIB has a better dispersion effect on carbon nanotubes. After the CNT is dispersed with the assistance of PIB-ED2003-PIB, it can effectively stabilize the CNT solution, and result in the hindrance of precipitation. Therefore, PIB-ED2003-PIB can be used as one of the effective dispersants of CNT. This is particularly true after the solution is left to stand for 20 days, as PIB-ED2003-PIB shows good stability in dispersing carbon nanotubes. The dispersed carbon nanotubes by the four types of dispersants at their respective optimal weight ratio are observed for their microstructures and the dispersion with a transmission electron microscope, as shown in Figure 1b. While the carbon nanotubes, without the dispersant added, are entangled and agglomerated, those with dispersants added show an improvement in the agglomeration, which is especially significant with PIB-ED2003-PIB. This is consistent with the above experimental results, and PIB-ED2003-PIB is, therefore, used as the main dispersant for subsequent experiments.

### 3.2. Preparation of Counter Electrodes with CNT/PIB-ED2003-PIB

**Figure 2:**
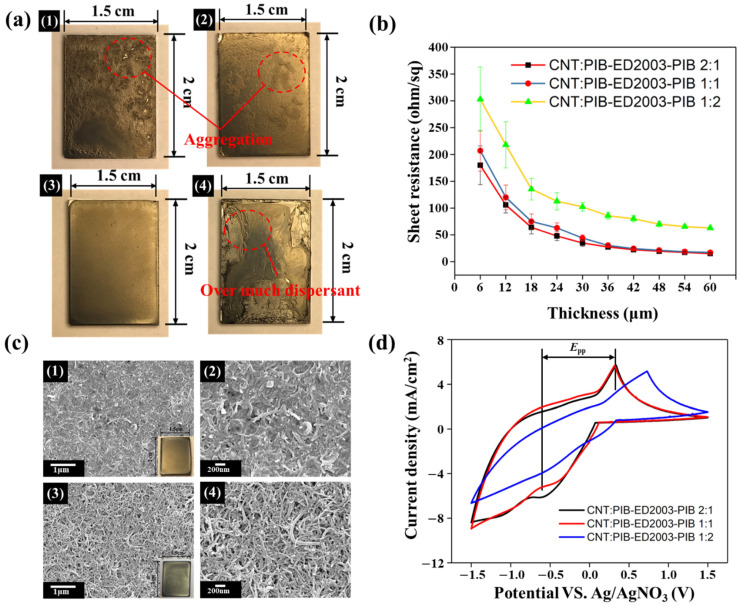
(**a**) Photos of the films created (1) without dispersant and when CNT:PIB-ED2003-PIB has a ratio of (2) 2:1, (3) 1:1, and (4) 1:2. (**b**) Resistance values with different PIB-ED2003-PIB contents and thicknesses. (**c**) Schematics (1,2) before and (3,4) after sintering of CNT:PIB-ED2003-PIB at weight ratio 1:1. (**d**) CV diagram of CNT-based counter electrode with PIB-ED2003-PIB added at different weight ratios.

As it is known that PIB-ED2003-PIB can effectively disperse carbon nanotubes, PIB-ED2003-PIB and carbon nanotubes will be used to prepare the counter electrode. First, 0.25 wt.% was applied to the counter electrode using a drop-coating method at 0.45 mL each time and then dried at 110 °C to form a film. This film formation process is observed macroscopically, as shown in Figure 2a. The results show that, without the dispersant, the counter electrode based on carbon nanotubes causes multiple locations of agglomeration that are distributed unevenly. With the dispersant added at a ratio of 2:1, agglomeration is still found at different places; only when the ratio is 1:1 does the agglomeration show good evenness; yet when the ratio is increased to 1:2, the excessive dispersant on the surface affects the overall integrity. Subsequently, when the ratio of CNT to PIB-ED2003-PIB is 1:1, the sheet resistance and thickness are measured using a four-point probe, as shown in Figure S6. As the number of layers and thus thickness increase gradually, the sheet resistance decreases from 206.7 to 12.7 Ω/sq., which reaches the limit between 12 and 15 Ω/sq. after 10 layers. Therefore, the subsequent preparation of the counter electrode will be based on 10 layers of coating as the standard. Following which, the sheet resistance with different weight ratios of CNT and PIB-ED2003-PIB is analyzed, as shown in Figure 2b. It can be observed that, as the ratio of dispersant increases, the resistance of the CNT-based counter electrode increases from 15.2 to 62.9 Ω/sq. This is because excessive dispersant wraps around the carbon nanotubes, which reduces the electron transferability. Therefore, considering the uniformity of the film and the sheet resistance, the weight ratio is determined to be 1:1 in future experiments. As the polymer-assisted dispersant in the final product gradually affects the counter electrode due to its poor conductivity, the pyrolysis temperature of PIB-ED2003-PIB is determined using TGA, as shown in Figure S7, to improve the conductivity of the counter electrode. The counter electrode is sintered to 350 °C for 1 h. The temperature is determined because the highest temperature of pyrolysis for the polymer-assisted organic dispersant is at 294.5 °C. SEM was used to observe the morphology of the counter electrode before and after sintering, as shown in Figure 2c. It can be observed that, before sintering, there are many uniformly distributed carbon nanotubes with one-dimensional structures on the surface of the counter electrode, which appear to be wrapped in polymer-assisted dispersant. After sintering, the CNT-based counter electrode has a clearer, rougher, and interlaced carbon nanotube structure on its surface. The sheet resistance is measured with a four-point carbon needle and recorded in Table 1. The sheet resistance of the counter electrode is detected to be 8.45 Ω/sq. after sintering, which is close to the value of 7 Ω/sq. for FTO glass. Therefore, to further study the electrocatalytic properties of CNT-based counter electrodes and the influences of different specific surface areas on the I^−^/I_3_^−^ redox couple, cyclic voltammetry (CV) is carried out, as shown in Figure 2d. The results show that carbon nanotubes have obvious wave patterns at both reduction and oxidation potentials, which means that the material has excellent redox properties. Although the waveforms are similar when the weight ratio is 2:1 and 1:1, the reduction potential is higher for the former than the latter, indicating that the reactivity of the I^−^/I_3_^−^ redox couple is higher when the weight ratio is 2:1. The CV data is summarized in Table 1. In contrast, the overpotential (ΔEp) is lower when the weight ratio is 1:1. As ΔEp is inversely proportional to the rate constant of the standard electrochemical redox reaction, it implies that a smaller ΔEp represents better catalytic activity. This also indicates that appropriately increasing the specific surface area of the CNT-based counter electrode creates more sites that are in contact with the I^−^/I_3_^−^ ions in the electrolyte, which helps in improving the catalytic activity of the I^−^/I_3_^−^ ions in the electrolyte and the capability to transfer electrons. In addition, when PIB-ED2003-PIB is added at a weight ratio of 1:2, the ΔEp and Ipc based on CV analysis both perform poorly. This indicates that, even if excessive dispersant is removed through the sintering process, it will nevertheless affect the content of the carbon nanotube layers, which, in turn, affects the integrity and redox properties of the film.

### 3.3. Preparation of the Highly Conductive CNT/PtNP Counter Electrode

**Figure 3:**
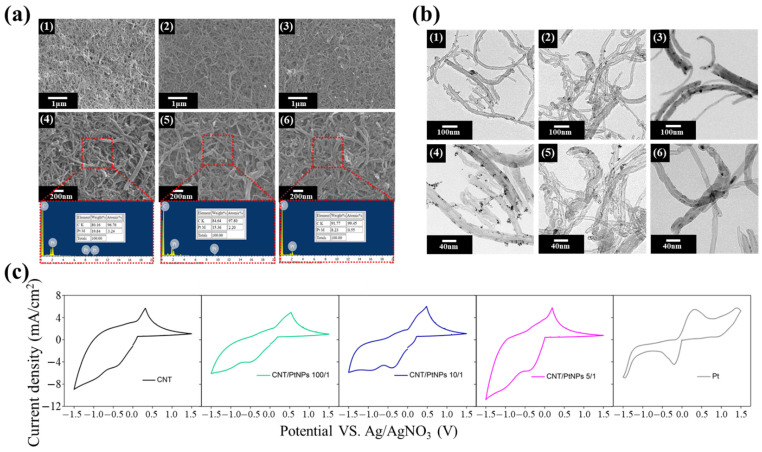
(**a**) SEM and EDS diagrams of CNT/PtNPs at different ratios: (1,4) 5/1 ratio, (2,5) 10/1 ratio, and (3,6) 100/1 ratio; (**b**) TEM diagrams of CNT/PtNPs at different ratios: (1,4) 5/1 ratio, (2,5) 10/1 ratio, and (3,6) 100/1 ratio; (**c**) Cyclic voltammetry spectra of CNT/PtNPs at different ratios.

It is known that the appropriate weight ratio of CNT:PIB-ED2003-PIB should be 1:1. The solution with this weight ratio is prepared, into which hexachloroplatinic acid is added. The solution is then reduced with sodium borohydride. SEM and EDS are used to observe the morphological changes and contents for the different ratios of platinum, as shown in Figure 3a. It can be observed that the CNT/PtNPs thin film has a rough surface composed of one-dimensional carbon nanotubes. However, it is not easy to observe the attachment of platinum nanoparticles. This is due to the size of the platinum nanoparticles, which is 1–3 nm, making it difficult for them to be observed using SEM. In contrast, the EDS elemental analysis reveals that the Pt content attached to the film increases as the weight ratio increases, indicating that the thin film contains platinum nanoparticles. Subsequently, the result of the synthesis is identified using the soft matter analysis through transmission electron microscope (TEM), as shown in Figure 3b. It can be found that after the synthesis of CNT/PtNPs, the platinum nanoparticles on the tubular carbon nanotube material are evenly attached to the walls of the carbon nanotubes. As the platinum content drops to 100/1, the attached nanoparticles also become less. The dispersant PIB-ED2003-PIB is added at a weight ratio of CNT/PIB-ED2003-PIB of 1:1, which stabilizes the carbon nanotubes in the solvent. At 0.25 wt.%, the NMP solution with the dispersant did not precipitate after standing for 20 days. The results of TEM and SEM reveal that the dispersant helps to disperse and stabilize the CNT/PtNPs hybrid material in the liquid phase. Furthermore, the four-point probe helps measure the sheet resistance of the film, as shown in Table 1. It can be seen that without an addition of platinum, the sheet resistance of CNT film alone reaches 8.45 Ω/sq., which is close to the 7 Ω/sq. of transparent conductive glass, indicating that CNT-based counter electrode film has the potential to replace FTO. In addition, as the content of platinum increases, the sheet resistance decreases accordingly, becoming even closer to that of the transparent conductive glass. This shows that the platinum nanoparticle not only increases the surface area, but also enhances the conductivity of the film after synthesis. Cyclic voltammetry is then used to analyze the CNT-based counter electrode with different ratios of platinum, as shown in Figure 3c. This result of CNT shows that the reduction in the current density is greater than the oxidation current density, which may be because CNTs can effectively promote the reduction reaction. Thus, the film that contains CNTs shows a higher reductive current density; this result is similar to other reports [46,47]. The data of all the samples are summarized in Table 1. The results show that adding a small amount of PtNPs does not lead to a significant improvement in Epp and Ipc for the CNT-based counter electrode, but rather a trend of deterioration. It is speculated that the NaBH4 added for the reduction process when synthesizing CNT/PtNPs may have affected the surface energy of CNTs, causing the reactivity to the electrolyte to reduce. This is why Epp and Ipc drop from 0.73 V and 4.75 mA cm^−2^ with pure CNT to 1.01 V and 3.96 mA cm^−2^ with CNT/PtNPs. Nevertheless, the trend is reversed when the ratio is changed from 10/1 to 5/1. In comparison to pure CNT, CNT/PtNPs at a ratio of 5/1 show a 30% improvement in Epp of the original value, and a 20% improvement in terms of Ipc, indicating that enough platinum nanoparticles can serve as the catalytic sites for carbon nanotubes, which improves the catalytic activity. At the same time, the CNT/PtNPs hybrid material is also compared with pure platinum. The data show that platinum has extremely obvious oxidation and reduction peaks, indicating that it has an excellent redox capability and is sensitive. However, in terms of Epp and Ipc, pure platinum does not perform as well as the CNT/PtNPs hybrid material at the optimal ratio of 5/1, the values of which are 0.55 V and 4.4 mA cm^−2^, respectively. This indicates that CNT/PtNPs can provide a higher current density for the catalysis of I_3_^−^; that is, CNT/PtNPs allow a higher catalytic current than Pt. In summary, by adding platinum nanoparticles that have excellent catalytic properties, carbon nanotubes also gain in specific surface area, exhibiting extremely excellent redox capabilities. As such, CNT/PtNPs may be a relatively suitable material for counter electrodes. Further, it is predicted that CNT/PtNPs at a ratio of 5/1 are the best for application to a counter electrode, while hybrid materials with ratios of 100/1 and 10/1, as well as pure CNT material, are less suitable. In addition, it is expected that the CNT/PtNPs hybrid material can surpass or approach the dye-sensitized solar cells formed by the platinum-based counter electrode in terms of efficiency.

### 3.4. Fabrication and Photoelectric Conversion Efficiency of DSSCs

**Figure 4:**
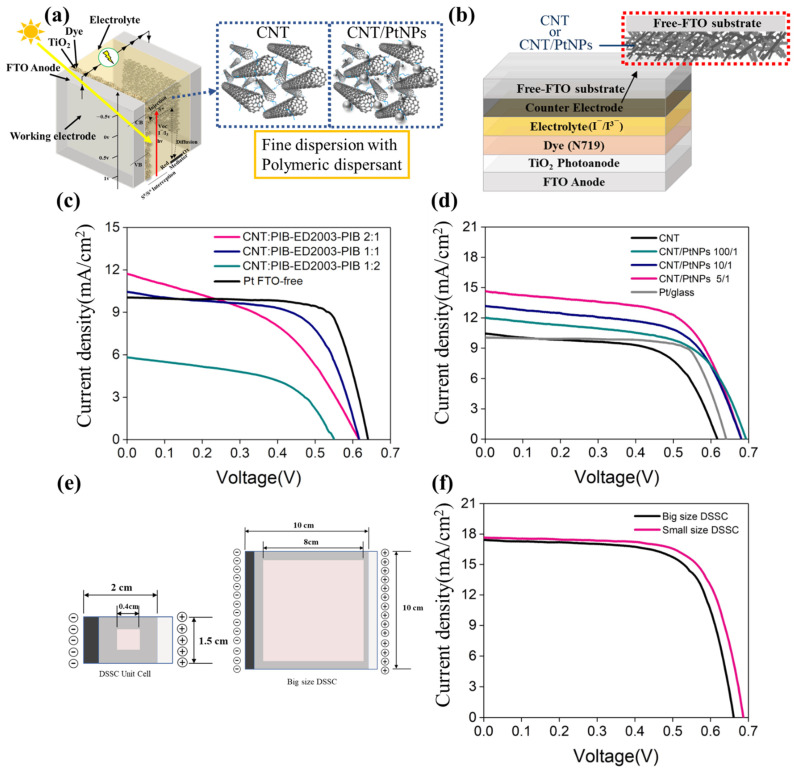
(**a**) DSSC packaging schematic. (**b**) DSSC cross-sectional view. (**c**) Specification diagrams of small-scale and large-scale DSSCs. (**d**) J–V curves of small-scale and large-scale DSSCs. (**e**) Schematic diagrams of small-scale (0.4 cm × 0.4 cm) and large-scale (8 cm × 8 cm) DSSCs. (**f**) Photoelectric conversion efficiencies of small-scale (0.4 cm × 0.4 cm) and large-scale (8 cm × 8 cm) DSSCs.

The above samples are packaged for dye-sensitized solar cells (DSSCs), as shown in Figure 4a,b, which are, respectively, the schematic diagram and cross-sectional view of the DSSC composition of the study. The counter electrode made without conductive glass for the DSSC with PIB-ED2003-PIB/carbon nanotubes at different ratios is first packaged into the cell, then placed under a solar simulator. The J–V curve is as shown in Figure 4c, and the data are summarized in Table 2. It can be observed that the maximum value of Jsc changes from 11.82 (at 2:1 ratio) to 10.4 (at 1:1 ratio) and finally to 5.84 (at 1:2 ratio). In comparison, the value is 10.04 for platinum on the glass. This implies that when the ratio of dispersant is increased to 1:2, the CNT-based counter electrode is not quite effective in catalyzing iodide ions, nor poses a strong resistance to the diffusion of iodide ions, and the difference in conductivity causes the conversion efficiency to be merely 1.83%, which is consistent with the prediction based on the above experimental results. In comparison, when the ratio is 2:1, Jsc shows a J–V curve that drops in value at an earlier stage, indicating that the FF value is relatively low at only 0.44. This shows that the counter electrode based on pure carbon nanotubes with a relatively small amount of dispersant does not show effective catalytic capability when in contact with the iodide-containing electrolyte. As a result, with a uniform dispersion process (at 1:1 ratio), the CNT-based counter electrode shows relatively good catalytic properties (FF of 0.63), which is close to that of platinum (fill factor (FF) of 0.68). Hence, its conversion efficiency of 4.03% is also close to the 4.37% of platinum, indicating that CNT can be considered a replacement for platinum. The highly conductive CNT/PtNPs counter electrode, fabricated by adding platinum, is packaged using the same method, the J–V curve obtained through measurement on non-conductive glass is shown in Figure 4d, and the data for analysis are summarized in Table 2. The results show that the data agree with the prediction results. At a ratio of 5/1, CNT/PtNPs achieves its optimal conversion efficiency of 6.28%. As the percentage of PtNPs increases, it can be observed that the short-circuit current density (Jsc) increases from the 10.40 mA cm^−2^ of pure CNT to the maximum value at 14.63 mA cm^−2^, while the FF is kept within the range of 0.60–0.63, indicating that the thin film is relatively uniform. Comparing the conversion efficiency of the counter electrode made of the film (4.37%) and pure platinum on non-conductive glass, it can be seen that the efficiency improves significantly, indicating that CNT/PtNPs is a potential material to replace platinum on non-conductive glass. Other than conducting the analysis of photoelectric conversion efficiency on non-conductive glass, the J–V curve of the DSSC composed of the same material on the traditional FTO is also discussed, as shown in Figure S8, and the data of which are summarized in Table 3. The results are similar to those of the experiments conducted on non-conductive glass. When the CNT/PtNPs ratio is 5/1, the maximum efficiency is 8.45%, which even exceeds the 8% efficiency of platinum on FTO. Moreover, the DSSC formed on FTO shows improvement in the open-circuit voltage (Voc), short-circuit current density (Jsc), and FF. This is because the FTO can provide a relatively better catalytic current in terms of conductivity, which also implies that the preparation on FTO still has certain advantages. To show the uniformity and stability of the counter electrode based on CNT/PtNPs hybrid film prepared by the organic dispersant in this study, a performance analysis is also carried out for a relatively large-scale DSSC, the preparation process of which is shown in Figure S9, with specifications in Figure 4e.

The J–V curves of the large-scale DSSC and the small-scale DSSC are compared, as shown in Figure 4f, and the data are summarized in Table S2. The results show that when the working area is increased to 8 cm × 8 cm, the efficiency of the DSSC is 7.95%—an overall 6% decrease compared to the 8.45% efficiency of the DSSC with a working area of 0.4 cm × 0.4 cm. This indicates that the organic/inorganic CNT/PtNPs hybrid film material prepared in this experiment has certain stability and can be used for the large-scale industrial production of DSSCs.

## 4. Conclusions

In this study, a counter electrode material without FTO glass was prepared successfully, which is proven to have a high photoelectric conversion efficiency in DSSCs. Carbon nanotube powder generally faces the problems of entanglement and aggregation in the liquid phase, which rely on the effects of Van der Waals force and hydrophobicity. These problems were improved with the use of polymer-assisted dispersants. The chemical structures of various dispersants were compared, including the differences in hydrophobic single/double-branch PIB segments, molecular weight, and hydrophilicity/hydrophobicity. Based on the comparison, PIB-ED2003-PIB, which has double branches with a large molecular weight and is relatively hydrophilic, was selected as the optimal dispersant, as it can effectively increase the dispersibility of CNTs. After further processing by solution drop coating, a CNT-based counter electrode without conductive glass (FTO) was prepared. This electrode has high roughness, which increases the contact area with the electrolyte so that it can effectively provide active sites to catalyze triiodide ions. Compared with the platinum counter electrode, it has similar photoelectric conversion efficiency, indicating that carbon nanotubes can replace platinum as a counter electrode material. Furthermore, carbon nanotubes were mixed with platinum nanoparticles to make a counter electrode material. The platinum nanoparticles can increase the catalytic current density of carbon nanotubes and then increase the specific surface area, reducing the diffusion resistance of the electrolyte and the overall sheet resistance of the film. However, it is necessary to find an optimal ratio of the platinum nanoparticles to add to achieve the best overall performance. With the aid of non-conductive glass, the photoelectric conversion efficiency of the DSSC made with carbon nanotubes/platinum nanoparticles with a weight ratio of 5/1 can achieve a conversion efficiency of 6.28%. This value increases to 8.45% with the aid of FTO, while the current density is 17.50 mA cm^−2^. This is higher than the 7.99% conversion efficiency achieved by the 90-nanometer platinum used in an FTO-based counter electrode. Furthermore, a large-scale DSSC was prepared, and its excellent stability was demonstrated. Therefore, the results of this study show that platinum nanoparticles have the potential to replace conductive glass (FTO).

## Figures and Tables

**Table 1:** CV analysis table of CNT/PIB-ED2003-PIB at different weight ratios.

Counter Electrode	E_pp_ (V) ^a^	I_pc_ (mA cm^−2^) ^b^	Sheet Resistance (ohm/sq.)
CNT:PIB-ED2003-PIB 2:1	0.93	6.09	--
CNT:PIB-ED2003-PIB 1:1	0.73	4.75	8.45
CNT:PIB-ED2003-PIB 1:2	1.23	3.55	--
CNT-ED2003/PtNPs 100/1 ^c^	1.01	3.93	8.35
CNT-ED2003/PtNPs 10/1 ^c^	0.89	4.97	7.59
CNT-ED2003/PtNPs 5/1 ^c^	0.51	5.70	7.25
Pt ^d^	0.58	4.40	--

^a^ peak-to-peak separation; ^b^ cathodic current density; ^c^ CNT-ED2003 represents the ratio of CNT to PIB-ED2003-PIB was 1:1; ^d^ Pt were fabricated by sputtered, for 90 nm thickness.

**Table 2:** Efficiencies of the counter electrodes made of dispersant/CNT at different weight ratios and CNT/PtNPs at different ratios without FTO substrate.

Counter Electrodes ^a^	V_OC_ (V)	J_SC_ (mA/cm^2^)	FF	η (%)
CNT:PIB-ED2003-PIB 2:1	0.62	11.82	0.44	3.22
CNT:PIB-ED2003-PIB 1:1	0.62	10.40	0.63	4.03
CNT:PIB-ED2003-PIB 1:2	0.55	5.84	0.57	1.83
CNT-PIB-ED2003-PIB/PtNPs 100/1 ^b^	0.69	11.94	0.60	4.94
CNT-PIB-ED2003-PIB/PtNPs 10/1 ^b^	0.68	13.32	0.61	5.52
CNT-PIB-ED2003-PIB/PtNPs 5/1 ^b^	0.68	14.63	0.63	6.28
Pt ^c^	0.64	10.04	0.68	4.37

^a^ Film thicknesses were around 55 μm, made by drop coating 10 times, with a 0.5-milliliter drop each time and heating under 110 °C; ^b^ CNT-PIB-ED2003-PIB represents the ratio of CNT to PIB-ED2003-PIB was 1:1; ^c^ Pt was fabricated by sputtered, for 90 nm thickness.

**Table 3:** Efficiency of counter electrodes made of dispersant/carbon nanotubes at different weight ratios and carbon nanotubes/platinum nanoparticles at different ratios under FTO substrate.

Counter Electrode ^a^	V_OC_ (V)	J_SC_ (mA/cm^2^)	FF	η (%)
CNT:PIB-ED2003-PIB 1:1	0.65	10.82	0.64	4.52
CNT-PIB-ED2003-PIB/PtNPs 100/1 ^b^	0.64	11.49	0.64	4.67
CNT-PIB-ED2003-PIB/PtNPs 10/1 ^b^	0.66	15.78	0.69	7.19
CNT-PIB-ED2003-PIB/PtNPs 5/1 ^b^	0.68	17.50	0.71	8.45
Pt ^c^	0.68	18.65	0.63	7.99

^a^ Film thickness was around 55 μm, made by drop coating 10 times, each time drop 0.5 mL and heating under 110 °C. ^b^ CNT-PIB-ED2003-PIB represents the ratio of CNT to PIB-ED2003-PIB was 1:1. ^c^ Pt was fabricated by sputtered, for 90 nm thickness.

## Data Availability

Not applicable.

## Supplementary Materials

The following are available online at https://www.mdpi.com/article/10.3390/polym13183103/s1, Figure S1. PIB-M1000 block copolymer synthesized from polyisobutylene-g-succinic anhydride and polyetheramine monoamine functional group Jeffamine M1000 through amination and imidization. Figure S2. PIB-imide-PIB triblock copolymer synthesized from polyisobutylene-g-succinic anhydride and polyetheramine diamine functional groups Jeffamine ED900, ED2003, D2000 through amination and imidation, Figure S3. FTIR spectra of reactions with (a) PIB-M1000, (b) PIB-ED900-PIB, (c) PIB-ED2003-PIB, and (d) PIB-D2000-PIB, Figure S4. Photos of solubility tests with (a) PIB-M1000, (b) PIB-ED900-PIB, (c) PIB-ED2003-PIB, and (d) PIB-D2000-PIB on (1) H_2_O, (2) DMF, (3) NMP, (4) EtOH, (5) Acetone, (6) MEK, (7) THF, and (8) Toluene, Figure S5. Influences of different dispersants on the penetration of solutions with different weight ratios of carbon nanotubes at a wavelength of 550 nm after (a) 2 days, (b) 20 days of storage and actual photos of the solutions. Figure S6. Corresponding resistance and thickness based on different numbers of coating layers, Figure S7. TGA and DTG curves of PIB-ED2003-PIB, Figure S8. J–V curves of counter electrodes made of carbon nanotubes/platinum nanoparticles at different ratios without FTO, and J–V curves of counter electrodes made of carbon nanotubes/platinum nanoparticles at different ratios with FTO, Figure S9. Schematic of large-scale (8 cm × 8 cm) DSSC fabrication, Table S1. Solubility test results of various dispersants in different solvents, Table S2. Data for efficiency analysis of DSSCs of different working areas, Video S1. Demonstration of the DSSCs based on polymer-assisted dispersion of PtNPs/CNTs nanohybrid films as FTO-free counter electrodes was used both indoors and outdoors.
