# Supplementary material for: Enhanced Efficiency of Dye-Sensitized Solar Cells Based on Polymer-Assisted Dispersion of Platinum Nanoparticles/Carbon Nanotubes Nanohybrid Films as FTO-Free Counter Electrodes
Source: Polymers (Basel). 2021 Sep 15;13(18):3103. doi: 10.3390/polym13183103 (PMC8469940; doi:10.3390/polym13183103)
Supplement: Supplementary file 1 [file polymers-13-03103-s001.zip › polymers-1376344-supplementary/Chiu-3 Supporting Information (polymers-1376344) 9-Sep⌐/2021.pdf]

polymers

Supporting Information

Enhanced Efficiency of Dye-Sensitized Solar Cells Based on Polymer-Assisted Dispersion of Platinum Nanoparticles/Carbon Nanotubes Nanohybrid Films as FTO-Free Counter Electrodes

Jia-Wun Li [1,†], Yu-Sheng Chen [1,†], Yan-Feng Chen [1], Jian-Xun Chen [1], Chung-Feng Jeffrey Kuo [1], Liang-Yih Chen [2], and Chih-Wei Chiu [1,*]

[1] Department of Materials Science and Engineering, National Taiwan University of Science and Technology, Taipei 10607, Taiwan
[2] Department of Chemical Engineering, National Taiwan University of Science and Technology, Taipei 10607, Taiwan

* Correspondence: cwchiu@mail.ntust.edu.tw; Tel.: +886-2-2737-6521
† These authors contributed equally to this work.

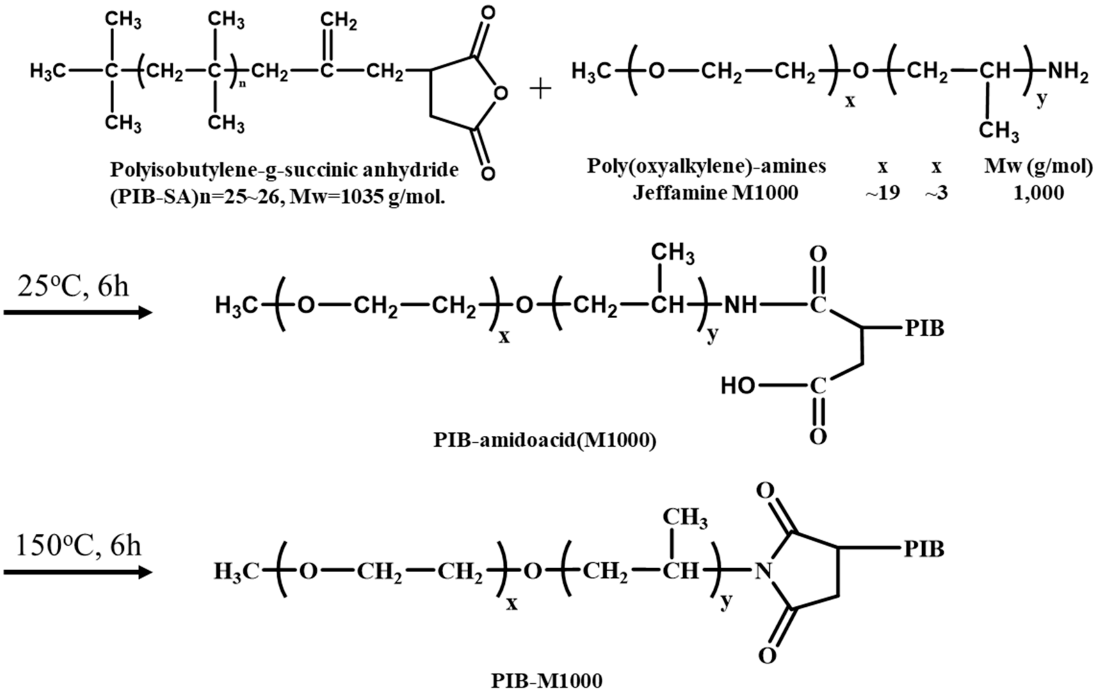

Figure S1. PIB-M1000 block copolymer synthesized from polyisobutylene-*g*-succinic anhydride and polyetheramine monoamine functional group Jeffamine M1000 through amination and imidization.

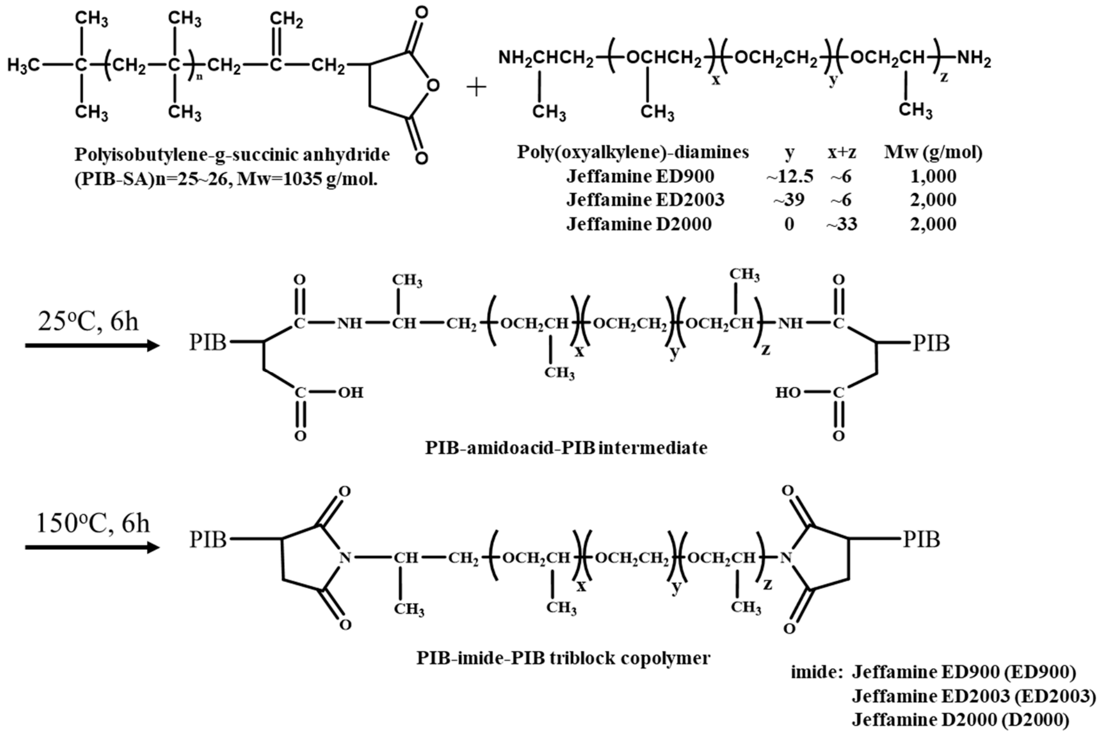

Figure S2. PIB-imide-PIB triblock copolymer synthesized from polyisobutylene-*g*-succinic anhydride and polyetheramine diamine functional groups Jeffamine ED900, ED2003, D2000 through amination and imidation.

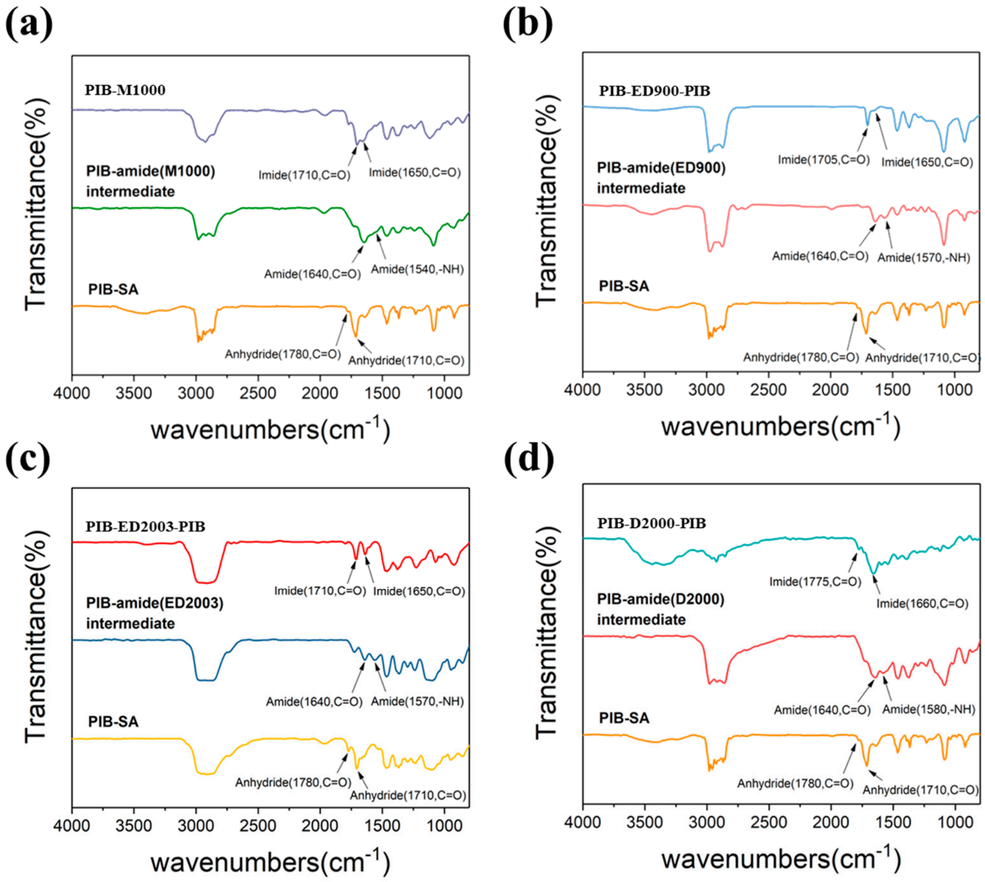

Figure S3. FTIR spectra of reactions with (a) PIB-M1000, (b) PIB-ED900-PIB, (c) PIB-ED2003-PIB, and (d) PIB-D2000-PIB.

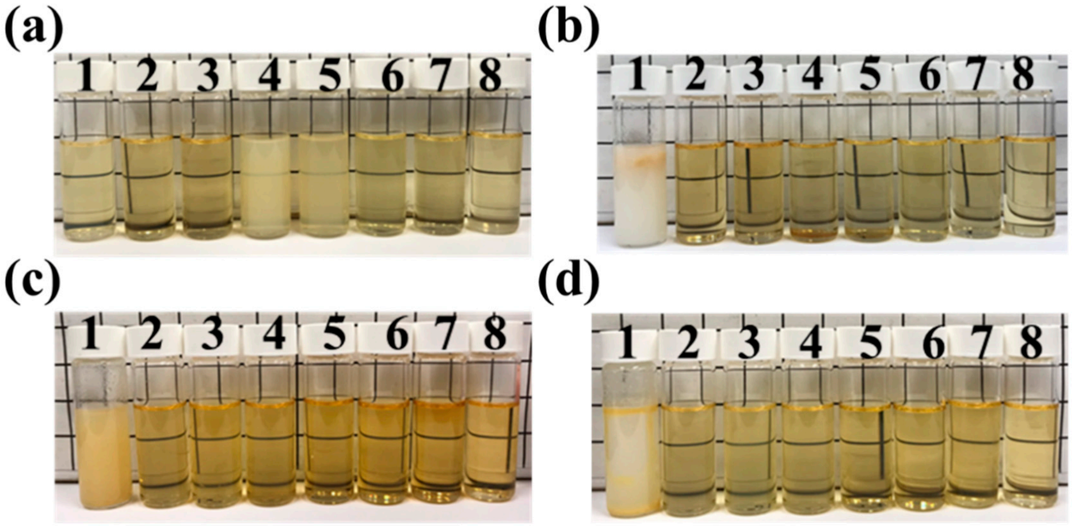

Figure S4. Photos of solubility tests with (a) PIB-M1000, (b) PIB-ED900-PIB, (c) PIB-ED2003-PIB, and (d) PIB-D2000-PIB on (1) H_2O, (2) DMF, (3) NMP, (4) EtOH, (5) Acetone, (6) MEK, (7) THF, and (8) Toluene.

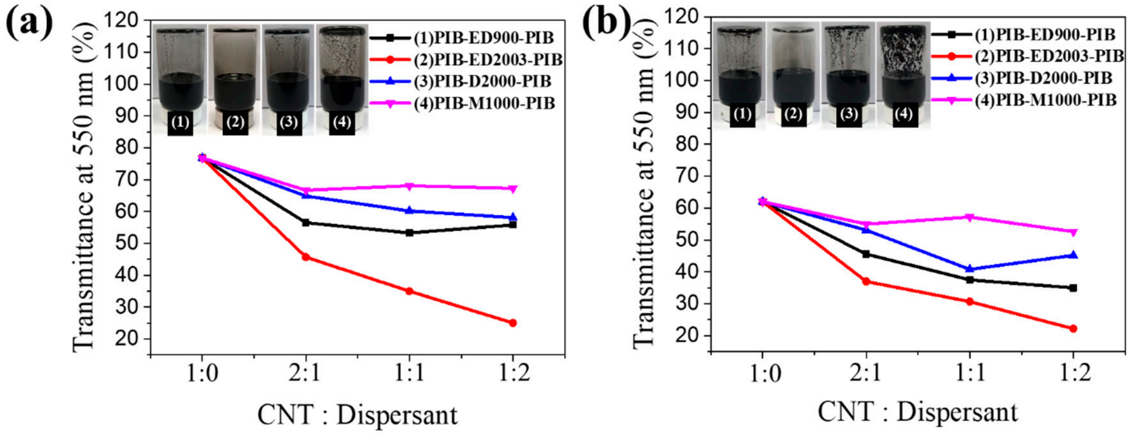

Figure S5. Influences of different dispersants on the penetration of solutions with different weight ratios of carbon nanotubes at a wavelength of 550 nm after (a) 2 days, (b) 20 day of storage and actual photos of the solutions.

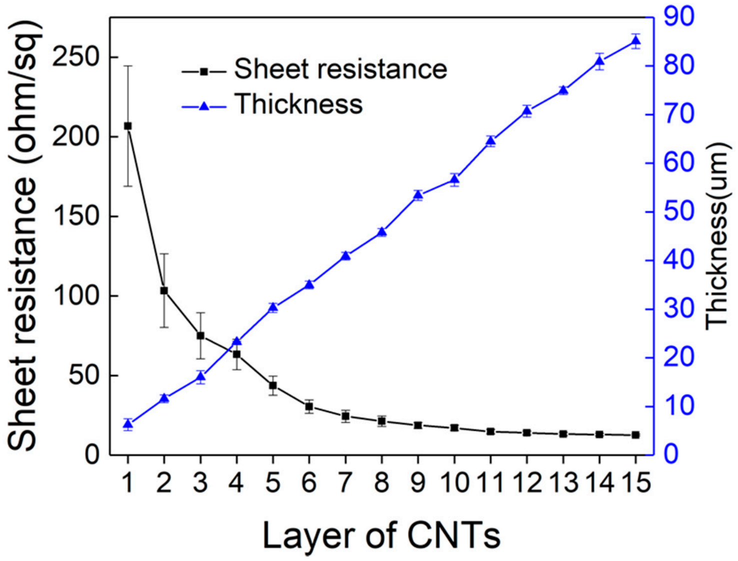

Figure S6. Corresponding resistance and thickness based on different numbers of coating layers.

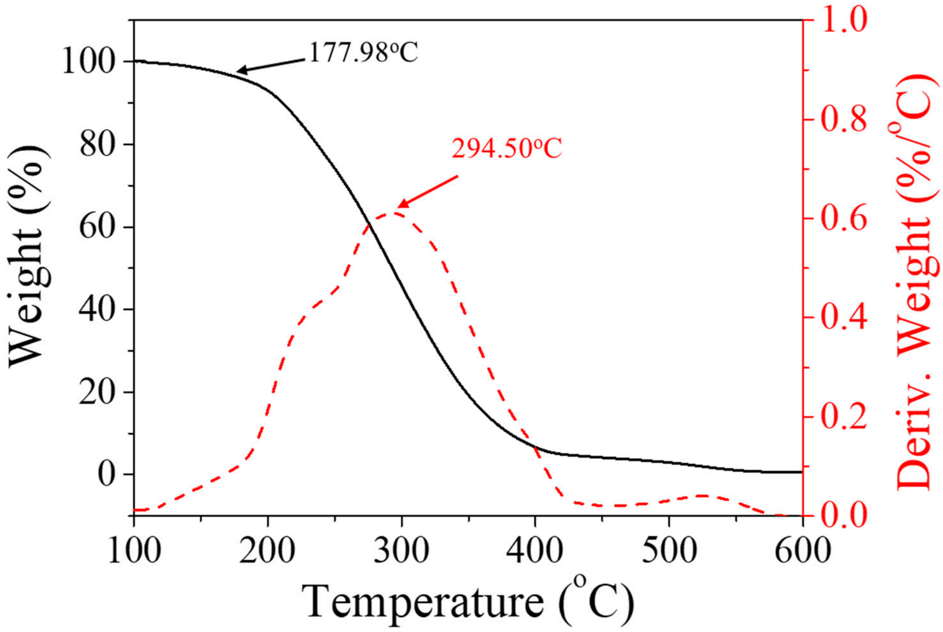

Figure S7. TGA and DTG curves of PIB-ED2003-PIB.

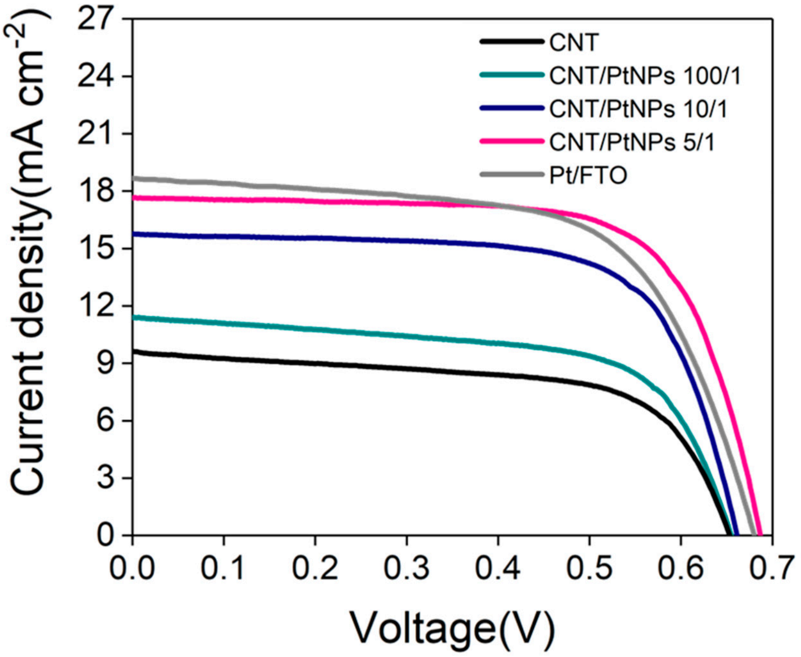

Figure S8. J-V curves of counter electrodes made of carbon nanotubes/platinum nanoparticles at different ratios without FTO, and J-V curves of counter electrodes made of carbon nanotubes/platinum nanoparticles at different ratios with FTO.

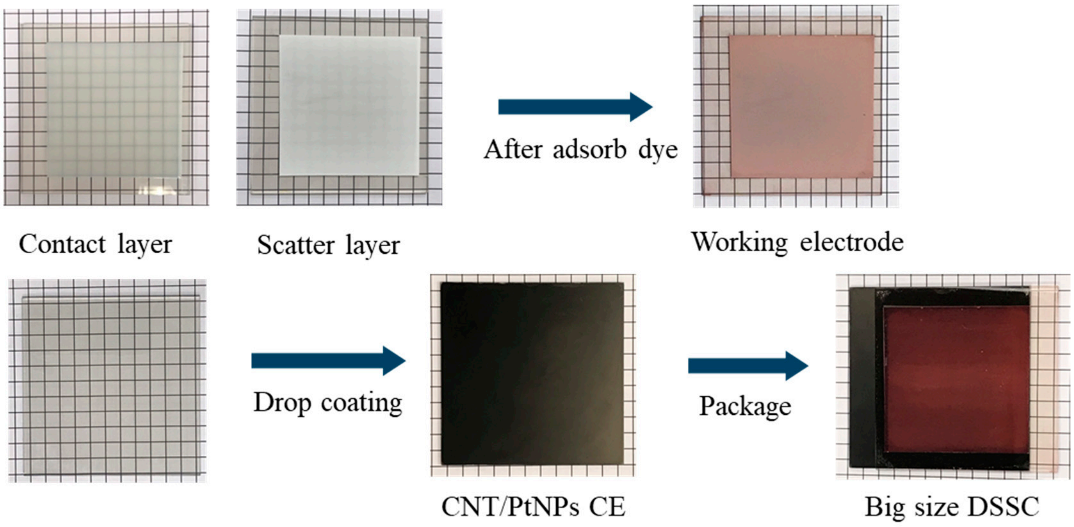

Figure S9. Schematic of large-scale (8 cm x 8 cm) DSSC fabrication.

Table S1. Solubility test results of various dispersants in different solvents.

Dispersant	Solvent[a]							
	H_2O	DMF	NMP	EtOH	Acetone	MEK	THF	Toluene
PIB-SA	-	-	+-	-	-	-	+	+
M1000	+	+	+	+	+	+	+	+
PIB-M1000	+	+	+	+	+	+	+	+
ED900	+	+	+	+	+	+	+	+
PIB-ED900-PIB	-	+	+	+-	+-	+	+	+
ED2003	+	+	+	+	+	+	+	+
PIB-ED2003-PIB	+-	+	+	+	+	+	+	+
D2000	-	+	+	+	+	+	+	+
PIB-D2000-PIB	-	+	+	+	+	+	+	+

[a] Concentration maintain in 0.2g /10 ml. +:soluble; +-:soluble but forming sediments after 2h sonication; -: insoluble.

Table S2. Data for efficiency analysis of DSSCs of different working areas.

DSCC	V_{OC} (V)	J_{SC} (mA/cm^2)	FF	η (%)
Small size DSSC[a]	0.68	17.50	0.71	8.45
Big size DSSC[b]	0.66	17.45	0.69	7.95

[a] working area = 0.4 cm × 0.4 cm.
[b] working area = 8 cm × 8 cm.
